# [Retracted] Embelin-induced brain glioma cell apoptosis and cell cycle arrest via the mitochondrial pathway

**DOI:** 10.3892/or.2023.8691

**Published:** 2023-12-27

**Authors:** Aiping Wang, Baochao Zhang, Jiandang Zhang, Wei Wu, Wei Wu

Oncol Rep 29: 2473–2478, 2013; DOI: 10.3892/or.2013.2369

Following the publication of the above paper, it was drawn to the Editor's attention by a concerned reader that the flow cytometric data shown in Fig. 4A on p. 2475 were strikingly similar to data appearing in another article written by different authors at different research institutes which had already been published.

Owing to the fact that the contentious data in the above article had already been published elsewhere prior to its submission to *Oncology Reports*, the Editor has decided that this paper should be retracted from the Journal. The authors were asked for an explanation to account for these concerns, but the Editorial Office did not receive a reply. The Editor apologizes to the readership for any inconvenience caused.

